# Longitudinal Analysis of the Intestinal Microbiota among a Cohort of Children in Rural and Urban Areas of Pakistan

**DOI:** 10.3390/nu15051213

**Published:** 2023-02-28

**Authors:** Veeraraghavan Balaji, Duy M. Dinh, Anne V. Kane, Sajid Soofi, Imran Ahmed, Arjumand Rizvi, Meera Chatterjee, Sudhir Babji, Joanne Duara, Joy Moy, Elena N. Naumova, Christine A. Wanke, Honorine D. Ward, Zulfiqar A. Bhutta

**Affiliations:** 1 Division of Geographic Medicine and Infectious Diseases, Tufts Medical Center, Boston, MA 02111, USA; 2 Department of Microbiology, Christian Medical College, Vellore 632004, India; 3 Division of Nutrition Data Sciences, Center of Excellence in Women and Child Health, The Aga Khan University, Karachi 74800, Pakistan; 4 Gerald J. and Dorothy R. Friedman School of Nutrition Science and Policy, Tufts University, Boston, MA 02111, USA; 5 Department of Public Health and Community Medicine, Tufts University School of Medicine, Boston, MA 02111, USA; 6 Centre for Global Child Health, The Hospital for Sick Children, Toronto, ON M5G 0A4, Canada; 7 Department of Nutritional Sciences, University of Toronto, Toronto, ON M5S 3E2, Canada

**Keywords:** intestinal, gut, microbiota, Pakistan, malnutrition

## Abstract

The profile of the intestinal microbiota is known to be altered in malnourished young children in low- and middle-income countries. However, there are limited studies longitudinally evaluating the intestinal microbiota in malnourished young children in resource-limited settings over the first two years of life. In this longitudinal pilot study, we determined the effect of age, residential location, and intervention on the composition, relative abundance, and diversity of the intestinal microbiota in a representative sample of children under 24 months of age with no diarrhea in the preceding 72 h in the urban and rural areas of Sindh, Pakistan nested within a cluster-randomized trial evaluating the effect of zinc and micronutrients on growth and morbidity (ClinicalTrials.gov Identifier: NCT00705445). The major findings were age-related with significant changes in alpha and beta diversity with increasing age. There was a significant increase in the relative abundance of the Firmicutes and Bacteroidetes phyla and a significant decrease in that of the Actinobacteria and Proteobacteria phyla (*p* < 0.0001). There were significant increases in the relative abundances of the major genera *Bifidobacterium, Escherichia/Shigella* and *Streptococcus* (*p* < 0.0001), and no significant change in the relative abundance of *Lactobacillus*. Using the LEfSE algorithm, differentially abundant taxa were identified between children in the first and second years of age, between those residing in rural and urban areas, and those who received different interventions at different ages from 3 to 24 months. The numbers of malnourished (underweight, wasted, stunted) or well-nourished children at each age, in each intervention arm, and at urban or rural sites were too small to determine if there were significant differences in alpha or beta diversity or differentially abundant taxa among them. Further longitudinal studies with larger numbers of well-nourished and malnourished children are required to fully characterize the intestinal microbiota of children in this region.

## 1. Introduction

Nutritional compromise, especially stunting among children under 5 years of age, remains a global problem, with an estimated 149 million children affected worldwide in 2021 and an additional 45 million children wasted [1]. Multiple factors likely contribute to the pathogenesis of linear growth retardation, including poor dietary intake, micronutrient deficiencies, multiple episodes of enteric or respiratory infections [2], or environmental enteric dysfunction studies suggest that intestinal microbiota contribute to intestinal epithelium proliferation and maturation, induction of genes for nutrient uptake, and development of the mucosal immune system, alongside an array of other metabolic, structural, and protective functions, regarding these as essential for optimum human health [3,4]. Given the temporal association of stunting in the first two years of life and its association with the frequent burden of infections [1], studies relating microbiome patterns to nutritional status during this sensitive period are critical to understanding the pathogenesis of undernutrition and potential therapies [5].

The gut microbiome has been increasingly implicated in malnutrition in young children in resource-limited settings (reviewed in [4,5,6,7,8,9,10,11,12]). Most of these studies have focused on severe acute malnutrition (SAM) or moderate acute malnutrition (MAM) [13,14,15,16,17,18,19,20,21]. A few studies have examined the gut bacteria and microbiota of young children with chronic malnutrition or linear growth stunting [15,22,23,24,25]. However, there are limited studies longitudinally evaluating the patterns of the intestinal microbiota in malnourished young children in resource-limited settings over the first two years of life.

We undertook a longitudinal study to determine the effect of age and residential location on the composition and diversity of the intestinal microbiota in children under 24 months of age in the urban and rural areas of Sindh, Pakistan nested within a cluster-randomized trial evaluating the effect of zinc and micronutrients on growth and morbidity (ClinicalTrials.gov Identifier: NCT00705445) [26]).

## 2. Materials and Methods

### 2.1. Study Design and Participants

This was a planned prospective pilot sub-study nested within a cluster-randomized trial of two micronutrient powder formulations with or without zinc, in children between the ages of 6 and 18 months in two representative populations to assess impact on growth and morbidity. The parent trial and characteristics of the population have been described in detail elsewhere [26]. Briefly, cluster randomization was used to generate three groups of children: Group A was the control group, Group B received a micronutrient powder (MNP) mixed with home-available food daily without zinc, and Group C received micronutrient powder with zinc (10 mg/day). A total of 256 clusters were identified: 145 from Matiari, a rural site about 200 km from Karachi, and 111 from Bilal colony, an urban squatter settlement in Karachi (See Figure 1 in [27]). A total of 2746 children entered the parent study at 3–6 months of age and were followed every three months until 24 months of age; home visitors collected information every two weeks from mothers about the presence or absence of diarrhea or respiratory symptoms. Anthropometric measures were obtained monthly using standard methods. The sub-study was planned on 60 consecutive children (with replacement for losses) for the assessment of serial intestinal microbiota in fecal samples to assess maturation patterns and the effect on nutrition interventions.

### 2.2. Fecal Sample Collection, DNA Extraction, Amplicon Generation, and 16S rRNA Sequencing

Fecal samples were routinely collected at baseline (3 months) as well as at 6, 9, 12, 18, and 24 months for enteric pathogens, ensuring the absence of diarrhea in the preceding 72 h. Once the subject was identified by the field teams in their weekly visits, a stool kit (comprising of diaper, spoon, container, and gloves) was provided to the family of the subject to collect a fresh fecal sample. A significant portion of the fecal sample was placed in the plastic screw-capped container using the disposable plastic spoon immediately after collection, ensuring the collection only of specimens not in contact with the diaper lining.

The collected fecal samples were transported in a special cold storage box with cold chain maintenance at a temperature of 2–8 °C within 6 h of collection from the field site to the Infectious Disease Research Laboratory (IDRL) at the Department of Pediatrics, Aga Khan University. In the research lab, these stool samples were stored at −80 °C. Ten children representing each of the three groups, from both urban and rural sites, and with fecal samples available at all of the time points (3, 6, 9, 12, 18, and 24 months), were randomly chosen for this sub-study. This provided a total of 360 fecal samples for the assessment of intestinal microbiota.

DNA was extracted from stool samples in the IDRL using a QIAamp Stool Mini kit (Qiagen, Inc., Valencia, CA, USA) and shipped to Tufts Medical Center in Boston. Upon receipt at Tufts Medical Center, DNA concentration was measured using a Nanodrop ND-1000 (Thermo Scientific, Wilmington, DE, USA) and normalized to 50 µg/mL in all samples by the addition of UltraClean PCR Water (MoBio, Thermo Fisher Scientific, Waltham, MA, USA). For sequencing runs performed on LR70 plates, (Roche, Indianapolis, IN, USA). PCR primers were designed to amplify the V4 hypervariable region of the genes for the 16S rRNA gene of both Bacteria and Archaea. The forward primer contained the V4 specific sequence AYTGGGYDTAAAGNG, an octamer barcode [28] unique to each sample to enable multiplexing, and the Roche adaptor sequence A. The reverse primer consisted of a pool of four V4 targeted sequences (TACCRGGGTHTCTAATCC, TACCAGAGTATCTAATTC, CTACDSRGGTMTCTAATC, and TACNVGGGTATCTAATCC) flanked by the Roche adaptor sequence B, used in the ratio 6:1:2:12, respectively. For titanium sequence runs, the primers were targeted to the V3-4 region. The reverse primer was a modified primer 926R containing the 454 Titanium Primer A sequence, a linker region (TCAG), and a unique 10-mer Hamady barcode sequence(′-CCATCTCATCCCTGCGTGTCTCCGACTCAG-XXXXXXXXXXCCGTCAATTCMTTTrelative abundance GT). The forward primer was a modified 357F primer containing the 454 Titanium adaptor B sequence and a linker (5′CCTATCCCCTGTGTGCCTTGGCAGTCTCAGCCTACGGGAGGCAGCAG).

PCR reactions were carried out in triplicate in parallel with a barcode-specific negative control; reactions yielding no amplicons or those in which the negative controls amplified were repeated. DNA concentration of amplicons was determined using a Quant-iT assay (Invitrogen, Carlsbad, CA, USA) and they were then pooled in equimolar concentrations. The amplicon pool was purified twice using an AMPure XP kit (Agencourt, Indianapolis, IN, USA) and sequenced on a Roche 454 Genome Sequencer GS FLX using the LR70 platform according to Roche protocols at the Virginia Bioinformatics Institute, and subsequently at the Tufts University Core Facility Genomics Core using a Roche 454 Genome Sequencer GS FLX+ and the Lib-L Sequencing kit according to Roche protocols. Replicates of the same sample were sequenced on both systems and the results showed no significant differences.

### 2.3. Computational and Bioinformatics Analyses

Computational analyses were performed using the open-source software platform QIIME Version 1.5 (http://qiime.org) [29]. The sequences were filtered for quality (minimum quality score = 25, minimum length 200, no ambiguous bases, no mismatches in primer) and assigned to samples by barcode. Similar sequences were clustered into operational taxonomic units (OTUs) based on a minimum identity of 97% using UClust [30]. The most frequent sequence within each OTU was used for alignment using PyNAST [29] and for the construction of a phylogenetic tree. This representative OTU was assigned to the lowest possible taxonomic level using the Ribosomal Database Project (RDP) Classifier (http://rdp.cme.msu.edu) [31]. The classification data were then used to generate comparisons of the relative abundance of selected phyla or genera between samples. The number of sequences was normalized and taxon-based alpha diversity measures including equitability, the number of observed species, Shannon diversity index, Chao-1, and phylogenetic-based alpha diversity measure PD (phylogenetic diversity) were determined.

### 2.4. Statistical Analysis

Statistical analyses were performed in R (http://www.r-project.org/). For categorical variables the difference was assessed with Fishers exact test while for continuous variables the difference was assessed with the Mann-Whitney test. Diarrhea was defined as three or more loose stools per day. A diarrheal episode was defined as a minimum of 2 days with diarrhea followed by at least 2 diarrhea-free days.

Beta diversity, defined as partitioning of diversity among communities, which is characterized using the number of species shared between communities, was determined by principal coordinates analysis (PCoA) of weighted UniFrac distances in QIIME based on age, residential location, and intervention arm. The ellipse R package, V3.3.2 was used to generate 95% confidence intervals and differences were determined using Adonis with 1000 permutations in the vegan R package V3.3.2. Temporal changes in overall alpha and beta diversity measures and taxon-specific relative abundances of the microbiota of all children in both locations and all three intervention arms were analyzed using a linear mixed-effects model (see below). Relative abundances for selected taxa were modeled as a function, of age adjusting for gender, residential location, and reported diarrheal episode.

To estimate the age-specific change in alpha diversity indices and relative abundance of major taxa and the degree of change at a group level, accounting for potential serial autocorrelation in the repeated measurements, the separate linear mixed-effects models were fitted to individual trajectories. Each model described outcomes as a function of time with age in days as a random variable and with gender, residential location, and diarrheal episode as fixed effect covariates using the lmer function of the lme4 R package, v3.3.2. Based on the model results, the 3-monthly rate of change for each outcome along with the lower and upper limits of their 95% confidence intervals (CI) were estimated.

The linear discriminant analysis effect size (LEfSe) algorithm was used to identify differentially abundant taxa in children in the first and second years of life combined or at each 3-monthly time point, by residential location and by intervention arms, using default parameters [32]. The relative abundance of each taxon for the longitudinal analysis was expressed as the percentage of the total for a given time point. Correction for multiple testing is not required for LEfSe [32]. As this was an exploratory study, corrections for multiple testing for other parameters were not performed [33].

## 3. Results

### 3.1. Clinical

Ten children from each of the three groups at both the rural and the urban sites were entered into this planned sub-study. Among the 60 sub-study sampled subjects, 9 (15%) children (7 from urban; 2 from rural) withdrew from the parent study before its completion. There was insufficient DNA from the stool of one child for sequencing; this child was also removed from the sub-study. Fecal samples for all of these dropped-out subjects were replaced by other randomly selected children. Thus, completion was assured for this sub-study, and all children enrolled had fecal samples from all six time points.

Table 1 shows the baseline statistics of subjects recruited from both urban and rural locations for this sub-study. 

**Figure 1:**
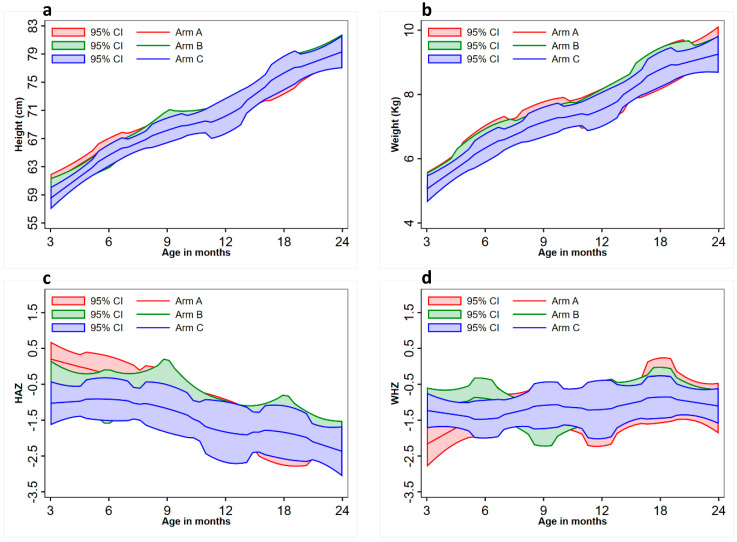
Growth trajectories of all children in different intervention arms: Mean (**a**) height, (**b**) weight, (**c**) HAZ (height for age) and (**d**) WHZ (weight for height) scores, and corresponding 95% CI (confidence intervals) for children in intervention arms A, B, and C from 3 to 24 months of age are shown.

The median age (in days) of children in the urban site was significantly higher (*p* < 0.001) than those in the rural site. The proportion of females in the rural site (43%) was higher than that in the urban site (30%). Proportions of wasted and stunted children in the recruited sample were similar across the urban and rural sites; however, the proportion of underweight children from the rural site was higher (47%) than that recruited from the urban site (23%). The median age of weaning (in months) was similar in both urban and rural localities. Detailed clinical data of all children at each time point are shown in Table S1 (Supplementary Data). Estimates on the nutritional indices revealed that, out of 60 children, 24 (40%) were wasted, 13 (22%) were stunted, and 21 (35%) were underweight at the time of recruitment. There was a gradual reduction in the proportion of wasted children over time, but stunting rates and the proportion of micronutrient deficiencies remained high. Overall, consonant with the findings from the main trial [26], there were no significant differences between the three groups for nutritional status at various time points, except for anemia and iron deficiency, which was lower in the two intervention arms at 18 months of age compared with the control group. Figure 1 shows that, while linear growth (HAZ scores) improved, WHZ scores remained unchanged overall.

Table 2 compares the clinical characteristics of the children in this subset for morbidities and antibiotic use at 3-monthly intervals over the study duration. 

A large proportion of children received antibiotics for common childhood illnesses (febrile episodes, fast breathing, and chest indrawing). As the stool microbiota sampling protocol precluded collection during diarrheal episodes, the subsample did not include children with diarrhea within 72 h of sample collection.

### 3.2. Gut Microbiota

**Figure 2:**
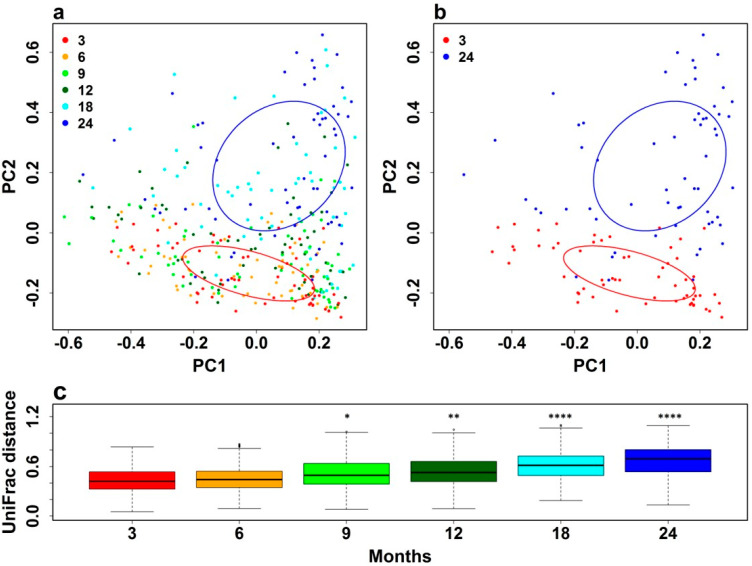
Beta diversity of the gut microbiota of all children: Beta diversity of the gut microbiota of all children was assessed using principal coordinates analysis (PCoA) of weighted Unifrac distances: (**a**) at 3-monthly intervals from 3 to 24 months and (**b**) at 3 and 24 months. (**c**) Comparison of weighted UniFrac distances at 6, 9, 12, 18, and 24 months compared with 3 months of age was determined using the ellipse R package, which was used to generate 95% confidence intervals. Differences were determined using Adonis with 1000 permutations in the vegan R package with Bonferroni correction. * *p* < 0.05, ** *p* < 0.01, **** *p* < 0.001.

Beta Diversity: We first assessed the beta diversity of the gut microbial community composition from all the children at each time point, in both locations (urban and rural) and within each intervention arm using PCoA of weighted UniFrac distances. The UniFrac algorithm measures similarity among microbial communities based on the degree to which their component taxa share branch length on a phylogenetic tree [34]. Figure 2a shows the clustering of gut microbial communities of all 60 children based on the age in months at which samples were collected and Figure 2b shows data from 3 and 24 months of age. 

Differences in weighted Unifrac distances between age groups were statistically significant at 9 (*p* < 0.05), 12 (*p* < 0.01), 18 (*p* < 0.001), and 24 (*p* < 0.001) months of age compared with 3 months of age (Figure 2c). There was no clustering by residential location (Figure S1) or intervention arm (Figure S2) and no significant differences in weighted Unifrac distance between children in urban or rural locations or in each intervention arm (not shown). These data indicate that, as expected [35], there were significant differences in gut microbial communities in the same children at increasing ages. However, there were no overall differences in beta diversity of gut microbial communities of children living in urban or rural locations or who received different interventions.

**Figure 3:**
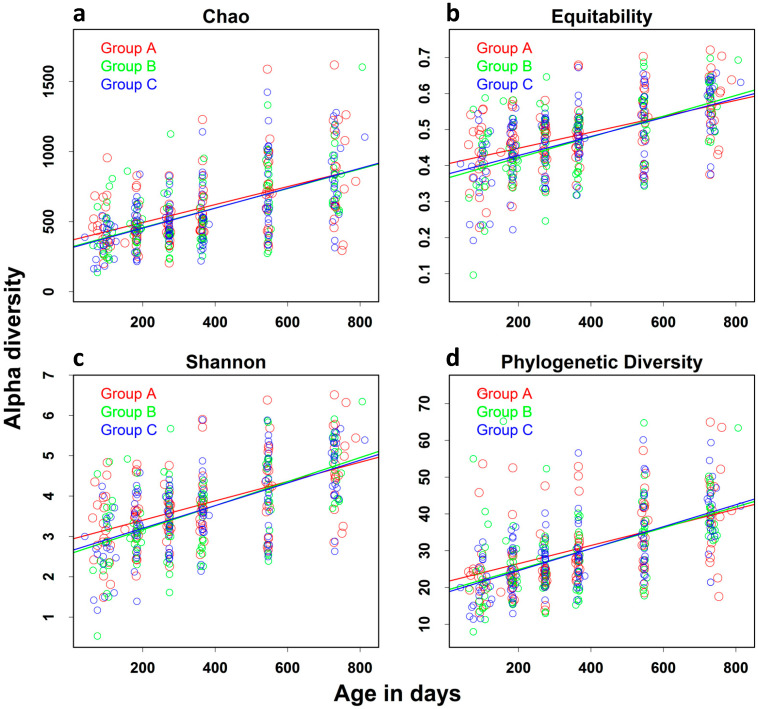
Effect of increasing age on alpha diversity: Alpha diversity of the gut microbiota of all children was assessed in QIIME Version 1.5 using taxon-based alpha diversity measures (**a**) Chao, (**b**) equitability, (**c**) Shannon diversity indices, and (**d**) phylogenetic-based alpha diversity measure phylogenetic diversity. The effect of increasing age on these alpha diversity measures was assessed using a linear mixed-effects model adjusted for gender, residential location, and reported diarrheal episodes.

Alpha Diversity: As age appeared to be the factor that most accounted for differences in beta diversity of microbial communities, we focused on the analysis of the effect of age on alpha diversity and relative abundance of major taxa at the highest and lowest taxonomic levels, i.e., phyla and genera. To account for covariates, we used a linear mixed-effects model adjusted for gender, residential location, and reported diarrheal episodes. Figure 3 shows a steady increase in four alpha diversity indices of all children in urban and rural locations combined with increasing age from 3 to 24 months. Using model estimation of the change in diversity indices associated with 3 months of age, we found that the rate of increase in alpha diversity was substantial and significant for all diversity indices examined (Table 3, *p* < 0.0001 for all indices). There was no significant difference in the rate of increase among children in the three intervention arms (not shown).

Although there were no significant differences overall in beta diversity of the gut microbiota of children residing in urban and rural locations, we wanted to determine if there were significant differences in alpha diversity indices between children residing in these locations at each of the 3-monthly time points. While all alpha diversity indices have doubled over the study period, as shown in Table S2 (Supplementary data), there were no significant differences in alpha diversity indices at any of the time points.

**Figure 4:**
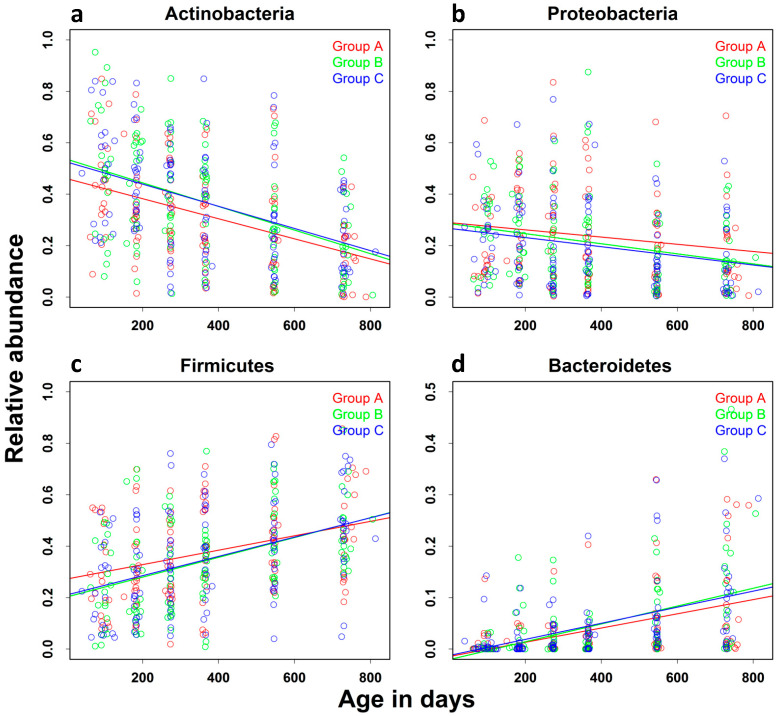
Effect of increasing age on the relative abundance of major taxa: The relative abundance of the four major taxa of the gut microbiota of all children, (**a**) Actinobacteria, (**b**) Proteobacteria, (**c**) Firmicutes, and (**d**) Bacteroidetes, was assessed in QIIME Version 1.5. The effect of increasing age on the relative abundance of these taxa was assessed using a linear mixed-effects model adjusted for gender, residential location, and reported diarrheal episodes.

Relative Abundance: Overall, the most abundant phyla were Actinobacteria, Firmicutes, Proteobacteria, and Bacteroidetes (Figure S3, Supplementary data). We performed a similar analysis for the effect of increasing age on the relative abundance of these four phyla, which were present in the microbiota of all of the children. There was a steady increase in the relative abundance of the Firmicutes and Bacteroidetes phyla and a decrease in that of the Actinobacteria and Proteobacteria phyla (Figure 4). The change in the age-related increase in the relative abundance of all four phyla was significant (shown as relative risk in Table 4, *p* < 0.0001 for all phyla).

**Figure 5:**
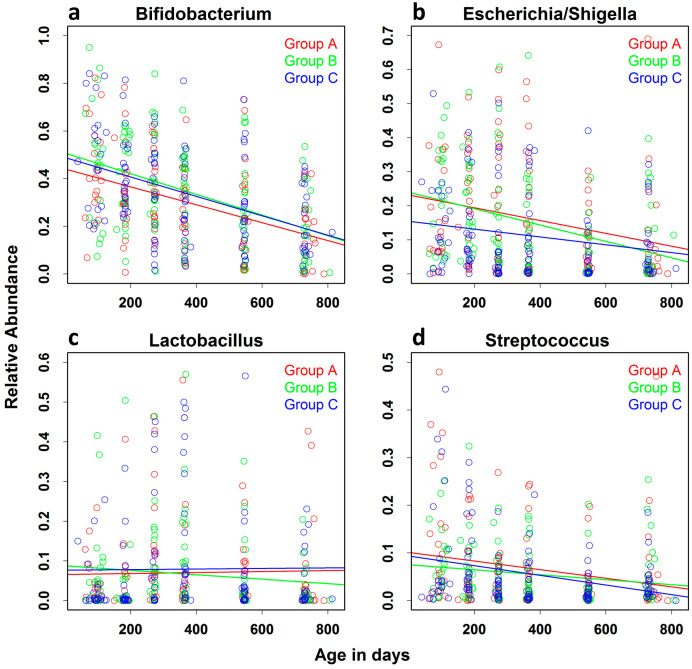
Effect of increasing age on the relative abundance of the most abundant genera: The relative abundance of the four most abundant genera of the gut microbiota of all children, (**a**) *Bifidobacterium*, (**b**) *Escherichia/Shigella* (**c**) *Lactobacillus*, and (**d**) *Streptococcus*, was assessed in QIIME Version 1.5. The effect of increasing age on the relative abundance of these genera was assessed using a linear mixed-effects model adjusted for gender, residential location, and reported diarrheal episodes.

Overall, the 10 most abundant genera were *Bifidobacterium*, *Escherichia*/*Shigella* (which cannot be distinguished from each other based on the sequence of the V4 region of the 16S rRNA gene amplicons), *Lactobacillus*, *Streptococcus*, *Prevotella*, *Actinobacillus*, *Fecalibacterium*, *Klebsiella*, and *Megasphera Lachnospiraceae* (Figure S4, Supplementary data). We determined the effect of increasing age on the relative abundance of four of the most abundant genera: *Bifidobacterium*, *Escherichia*/*Shigella*, *Streptococcus*, and *Lactobacillus*, which were present in the microbiota of all of the children (Figure 5). There was a steady decrease in the relative abundance of *Bifidobacterium*, *Escherichia*/*Shigella*, and *Streptococcus* and no apparent change in the relative abundance of *Lactobacillus* with increasing age. The change in the rate of decrease in the relative abundance of *Bifidobacterium* (*p* < 0.0001) *Escherichia*/*Shigella* (*p* < 0.0001), and *Streptococcus* (*p* < 0.0003) was highly significant, with no significant change in the relative abundance of *Lactobacillus* (Table 5). There was no significant difference in the rate of change in the relative abundance of these genera among children in each intervention arm (not shown). 

**Figure 6:**
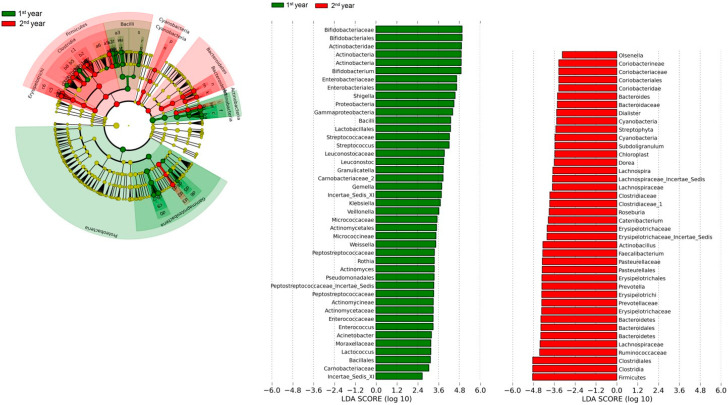
Differentially abundant taxa in the first and second years of life: Differentially abundant taxa at all taxonomic levels between the gut microbiota of all children in rural and urban locations in all intervention arms combined between the first and second years of life were assessed using the LEfSe algorithm.

Differentially abundant taxa: In order to identify differentially abundant taxa at all taxonomic levels between the microbiota of all of the children in rural and urban locations in all intervention arms combined between the first and second years of life, we used the LEfSe algorithm, which identifies genomic features characterizing the differences between two or more biological conditions [32]. This analysis (Figure 6) revealed that the microbiota of children in the first year of life were enriched in Actinobacteria and Proteobacteria and related taxa at different taxonomic levels in these phyla, whereas those of children in the second year of life were enriched in the Firmicutes, Bacteroidetes, and Cyanobacteria phyla and related taxa. For example, the related taxa Enterobacteriaceae, Enterobacteriales, Gammaproteobacteria, and *Shigella*/*Escherichia* in the Proteobacteria phyla were enriched in the first year of life.

We also used the LEfSe algorithm to identify differentially abundant taxa at all taxonomic levels between the microbiota of children in the urban and rural locations and in the three intervention arms, in each age group. These analyses revealed specific taxa that were enriched in the microbiota of children in urban versus rural locations in all intervention groups combined (Table S3, Supplementary data) and in each intervention arm in both urban and rural locations combined (Table S4, Supplementary data) at each age group. At baseline (3 months), the microbiota of children in the rural compared with urban locations were enriched in taxa in the Clostridiaceae, Lachnospiraceae, and Corynebacteriaceae families, as well as the Alphaproteobacteria class. At 6 months of age, when the interventions were started, Clostridiaceae and related taxa in the Firmicutes phylum continued to be enriched in the microbiota of children in the rural area, whereas the microbiota of children in the urban area were enriched in Actinobacteria and related taxa, including *Bifidobacterium*. However, by 9 months, there were no taxa enriched in children in the rural area, whereas the microbiota of children in the urban area were enriched in taxa in the Pasteurellaceae family. At 12 months, taxa in the Veillonellaceae family were enriched in the rural area. At 18 months of age, when the interventions were stopped, microbiota of children in the rural area were enriched in Enterococcaceae, Ruminococcaceae, and Ruminococcaceae_Incertae_Sedis families, whereas those in the urban area were enriched in taxa in the Erysipelotrichaceae family. By 24 months of age after 6 months of no intervention, only the *Sporobacater* genus was enriched in children in the rural area whereas the microbiota of children in the urban area were enriched in the Cyanobacteria phylum, the Clostridiaceae family, and the *Leuconostoc* genus.

There were very few differentially abundant taxa in the microbiota of children who received different interventions (Table S4, Supplementary data). At baseline (3 months), no specific taxa were enriched in any of the three intervention arms. At 6 months, when the interventions were started, taxa in the Actinomycetaceae family were enriched in children who received intervention C (micronutrient powder with zinc) compared with the control arm (A) and intervention arm B (micronutrient powder without zinc).

The numbers of malnourished (underweight, wasted, stunted) or well-nourished children at each age, in each intervention arm, and at urban or rural sites were too small to determine if there were significant differences in alpha or beta diversity or differentially abundant taxa among them.

## 4. Discussion

In this pilot study, we investigated the nutritional status and composition, relative abundance, and diversity of the gut microbiota of children in urban and rural areas of Pakistan from 3 to 24 months of age. The most significant findings were age-related changes with a decrease in HAZ score, increase in alpha and beta diversity, and changes in relative abundance of the major taxa at the level of phylum and genus with increasing age from 3 to 24 months of age. Differentially abundant taxa were identified between all of the children in the first and second years of life, between those residing in rural and urban areas, and those who received different interventions at different ages from 3 to 24 months.

Several studies, many of them longitudinal, have investigated the gut microbiota using 16S rRNA sequencing in children under the age of five years in low- and middle-income countries [15,16,18,20,22,23,25,35,36,37,38,39,40,41,42,43,44,45,46]. The type of study, methodology, sequencing platform, health status, age, diet, nutritional status, and geographic location of the children in these studies varied; therefore, comparisons between studies are difficult. However, longitudinal studies have established that the gut microbiota of infants and young children change considerably till 2 to 3 years of age [47]. In this study too, we found that increasing age had the greatest impact on the gut microbiota. Weighted Unifrac distances steadily increased and microbial communities clustered by age, indicating significant differences in beta diversity. All four alpha diversity indices also significantly increased with increasing age.

In the present study, we found that the most abundant phyla were Actinobacteria, Firmicutes, Proteobacteria, and Bacteroidetes, in descending order. This pattern of relative abundance of the major phyla differs by country. For example, in a similar longitudinal study from South India, Firmicutes and Proteobacteria were most abundant, followed by Actinobacteria and Bacteroidetes [22]. This may reflect differences in methodology such as sequencing platform, but may also be due to differences in the population studied.

Increasing age also had an impact on the relative abundance of the major Phyla, with significant increases in Firmicutes and Bacteroidetes and significant decreases in Actinobacteria and Proteobacteria with increasing age. Similarly, there were changes in the relative abundance of the four most abundant genera, with significant increases in *Bifidobacterium*, *Escherichia*/*Shigella*, and *Streptococcus* and no change in Lactobacillus with increasing age. In this study, using the LEfSe algorithm, we also found that Actinobacteria, Proteobacteria, and related taxa in the gut microbiota were enriched in the first year of life, compared with Firmicutes, Bacteroidetes, and Cyanobacteria and related taxa in the second year.

In the present study, we did not investigate eukaryotic components of the gut microbiota. Recently, Popovic et al., analyzed stool samples and data from a subset of 80 children at 12 and 24 months of age from the same clinical trial (ClinicalTrials.gov Identifier: NCT00705445) [26] as the present study to investigate the bacterial, protozoal, fungal, and helminth communities in the microbiota [27]. Although the sequencing platforms and microbiota analytic methods were different, they found, as did we, that Actinobateria and Firmicutes were the dominant phyla; that age was the main determinant of alpha and beta diversity; and that there was no association between nutritional status, location, or taxonomic composition. However, they did find that bacterial diversity was decreased in undernourished (WLZ < −2) children and that micronutrient supplementation was associated with specific bacterial communities. Most interestingly, they reported that, while eukaryotic communities, unlike bacterial communities, were not impacted by age, vitamin and iron supplementation was associated with colonization with specific protozoal and fungal communities, which was alleviated by zinc supplementation. In addition, children residing in the rural location had an increased prevalence of *Cryptosporidium* sp.

The age-related changes in the gut microbiota in the current study were similar to those from a longitudinal pilot study comparing low birth weight stunted children to normal birth weight non-stunted children from birth to 2 years of age in South India [22]. As in the current study, there were significant increases in alpha diversity indices and a relative abundance of Bacteroidetes and a decrease in that of Proteobacteria and Actinobacteria with increasing age in all of the children.

Specific taxa associated with linear growth stunting of children in longitudinal studies vary with geographic location, age, and analytic techniques. In the longitudinal study from India referred to above [22], the gut microbiota of stunted children were enriched in taxa in the *Desulfovibrio* genus and Campylobacterales order. In children from Malawi and Bangladesh, the relative abundance of *Acidaminococcus* sp. was associated with future deficits in linear growth [48]. In a study of young Bangladeshi children with linear growth stunting, Chen et al., found that the levels of a shared group of 14 bacterial taxa from duodenal aspirates were negatively correlated with linear growth [24]. These taxa included *Veillonella* sp., *Streptococcus* sp., and *Rothia mucilaginosa*.

Zinc deficiency has been shown to be associated with alteration in the gut microbiota in school-age children [49]. However, we did not find any differences in the composition or diversity of the gut microbiota in the intervention arm receiving MNPs containing zinc.

There are a few limitations to our pilot study. The numbers of well-nourished and malnourished children at each age, in each location, and intervention arm were small. We did not account for antibiotic use, diarrheal episodes, or diet, all of which are known to impact the gut microbiota. We used the Roche 454 platform for 16S rRNA sequencing, which was what was available at the time. In future studies, we will use the Illumina MiSeq platform for 16S sequencing and will also perform whole genome shotgun sequencing to characterize the gut microbiome of a percentage of subjects. The study design of the parent study did not include a cohort of healthy children. In future studies, we will also use the “relative microbiota maturity index” and “microbiota-for-age Z score” developed by Subramanian et al. [15] to assess the microbiota maturity of malnourished compared with healthy children of the same age.

This study was nested within a larger cluster-randomized trial on the effect of zinc and micronutrients on growth and morbidity. Consonant with the findings of the larger main trial [26] and the cross-sectional evaluation of a subgroup by Popovic et al. [27], the current study also does not show any differences between the groups for growth and nutritional outcomes. In contrast to the findings by Popovich et al. [27], no excess of pathogens was found in either of the two intervention arms in this study.

Further studies of larger numbers of malnourished (including underweight, wasted, and stunted) and well-nourished children using additional analytic tools such as “relative microbiota maturity index” and “microbiota-for-age Z score”, as well as metagenomics and metabolomics, are needed to identify differences in the gut microbiota between them and to design microbiota-directed interventions.

## Figures and Tables

**Table 1:** Baseline Data for Children in Urban and Rural Locations.

	Urban (*n* = 30)	Rural (*n* = 30)	*p* Value
Median Age (days, range)	101 (88–126)	91 (40–121)	0.0011 ^1^
Male (Number (percent)	9/30 (30%)	13/30 (43%)	0.422 ^2^
Wasted (percent)	13/30 (43%)	11/30 (37%)	0.7925 ^2^
Stunted (percent)	6/30 (20%)	7/30 (23%)	>0.999 ^2^
Underweight (percent)	7/30 (23%)	14/30 (47%)	0.1033 ^2^
Median Age of Weaning (months, range)	3 (1–4)	3 (0–6)	0.1075 ^1^

^1^ Mann-Whitney, ^2^ Fisher’s Exact Test.

**Table 2:** Clinical data of children in urban and rural locations in each intervention arm at 3 monthly intervals. (summary statistics reflect the count and percentages in parenthesis for categorical variables, while for continuous variables these are presented as median and IQR)

Parameter	Intervention Group			*p*-Value	Residence		*p*-Value
	A (*n* = 20)	B (*n* = 20)	C (*n* = 20)		Urban (*n* = 30)	Rural (*n* = 30)	
Weaning Age (months)	4.0 (4.0, 5.0)	5.0 (5.0, 6.0)	5.0 (4.5, 5.5)	0.058	5.0 (4.0, 5.0)	5.0 (5.0, 6.0)	0.140
At Recruitment (3 months)							
Breast Feeding	19 (95.0)	19 (95.0)	19 (95.0)	1.000	27 (90.0)	30 (100.0)	0.240
Animal Milk intake	2 (10.0)	2 (10.0)	5 (25.0)	0.470	8 (26.7)	1 (3.3)	0.026
Formula Milk intake	5 (25.0)	8 (40.0)	7 (35.0)	0.700	6 (20.0)	14 (46.7)	0.054
Antibiotic Use	3 (15.0)	5 (25.0)	8 (40.0)	0.240	4 (13.3)	12 (40.0)	0.039
Fever	7 (35.0)	8 (40.0)	10 (50.0)	0.720	8 (26.7)	17 (56.7)	0.035
Fast Breathing	5 (25.0)	5 (25.0)	1 (5.0)	0.190	0 (0.0)	11 (36.7)	<0.001
Chest Indrawing	2 (10.0)	2 (10.0)	0 (0.0)	0.530	2 (6.7)	2 (6.7)	1.00
At 6 months							
Breast Feeding	18 (90.0)	19 (95.0)	17 (85.0)	0.860	26 (86.7)	28 (93.3)	0.670
Animal Milk intake	2 (10.0)	2 (10.0)	4 (20.0)	0.710	7 (23.3)	1 (3.3)	0.052
Formula Milk intake	8 (40.0)	11 (55.0)	10 (50.0)	0.730	10 (33.3)	19 (63.3)	0.038
Weening food intake	17 (85.0)	18 (90.0)	16 (80.0)	0.900	28 (93.3)	23 (76.7)	0.150
Antibiotic Use	8 (40.0)	5 (25.0)	7 (35.0)	0.700	10 (33.3)	10 (33.3)	1.000
Fever	11 (55.0)	10 (50.0)	12 (60.0)	0.950	13 (43.3)	20 (66.7)	0.120
Fast Breathing	5 (25.0)	7 (35.0)	5 (25.0)	0.820	5 (16.7)	12 (40.0)	0.084
Chest Indrawing	0 (0)	0 (0)	0 (0)	--	0 (0)	0 (0)	--
Antibiotic Use	5 (25.0)	8 (40.0)	11 (55.0)	0.170	8 (26.7)	16 (53.3)	0.064
Fever	5 (25.0)	10 (50.0)	11 (55.0)	0.140	11 (36.7)	15 (50.0)	0.430
Fast Breathing	2 (10.0)	4 (20.0)	4 (20.0)	0.750	0 (0.0)	10 (33.3)	<0.001
Chest Indrawing	0 (0)	0 (0)	0 (0)	--	0 (0)	0 (0)	--
At 12 months							
Antibiotic Use	8 (40.0)	4 (20.0)	8 (40.0)	0.340	5 (16.7)	15 (50.0)	0.013
Fever	9 (45.0)	7 (35.0)	12 (60.0)	0.320	14 (46.7)	14 (46.7)	1.000
Fast Breathing	2 (10.0)	4 (20.0)	7 (35.0)	0.190	4 (13.3)	9 (30.0)	0.210
Chest Indrawing	0 (0.0)	1 (5.0)	2 (10.0)	0.770	0 (0.0)	3 (10.0)	0.240
At 18 months							
Antibiotic Use	8 (40.0)	4 (20.0)	7 (35.0)	0.470	9 (30.0)	10 (33.3)	1.000
Fever	9 (45.0)	8 (40.0)	12 (60.0)	0.520	8 (26.7)	21 (70.0)	0.002
Fast Breathing	2 (10.0)	7 (35.0)	4 (20.0)	0.190	2 (6.7)	11 (36.7)	0.010
Chest Indrawing	0 (0)	0 (0)	0 (0)	--	0 (0)	0 (0)	--
At 24 months							
Antibiotic Use	1 (5.0)	1 (5.0)	4 (20.0)	0.34	2 (6.7)	4 (13.3)	0.67
Fever	1 (5.0)	3 (15.0)	1 (5.0)	0.60	2 (6.7)	3 (10.0)	1.00
Fast Breathing	0 (0.0)	1 (5.0)	0 (0.0)	1.00	0 (0.0)	1 (3.3)	1.00
Chest Indrawing	0 (0)	0 (0)	0 (0)	--	0 (0)	0 (0)	--

**Table 3:** Relationship of Age and Alpha Diversity Indices.

Index	Age-Specific Effect * (SE)	LCI	UCI	*p* Value
Chao	60.627 (4.674)	51.462	69.792	<0.0001
Equitability	0.024 (0.0015)	0.018	0.027	<0.0001
Shannon	0.081 (0.005)	0.069	0.092	<0.0001
PD	2.427 (0.195)	2.046	2.811	<0.0001

* Expressed as age-specific effect associated with three months of age, adjusted for covariates (gender, urban/rural location, diarrheal episode) PD, phylogenetic diversity.

**Table 4:** Relationship of Age and Relative Abundance of Major Phyla.

Relative Abundance	Age-Specific Effect * (SE)	LCI	UCI	*p* Value
Actinobacteria	−0.039 (0.003)	−0.048	−0.033	<0.0001
Bacteroidetes	0.015 (0.0015)	0.012	0.018	<0.0001
Firmicutes	0.030 (0.003)	0.024	0.039	<0.0001
Proteobacteria	−0.015 (0.003)	−0.021	−0.006	0.0001

* Expressed as age-specific effect associated with three months of age, adjusted for covariates (gender, urban/rural location, diarrheal episode).

**Table 5:** Relationship of Age and Relative Abundance of Major Genera.

Relative Abundance	Age-Specific Effect * (SE)	LCI	UCI	*p* Value
*Bifidobacterium*	−0.0372 (0.0041)	−0.0454	−0.0291	<0.0001
*Shigella/Escherichia*	−0.0157 (0.0032)	−0.0221	−0.0095	<0.0001
*Lactobacillus*	−0.0006 (0.0025)	−0.0057	0.0043	0.7950
*Streptococcus*	−0.0077 (0.0021)	−0.0119	−0.0035	0.0003

* Expressed as age-specific effect associated with three months of age, adjusted for covariates (gender, urban/rural location, diarrheal episode).

## Data Availability

The de-identified data for the main study is available through the Ki initiative at the Bill & Melinda Gates Foundation. Additional microbiota data can be requested from the corresponding authors.

## Supplementary Materials

The following supporting information can be downloaded at https://www.mdpi.com/article/10.3390/nu15051213/s1. Figure S1. Beta Diversity of the gut microbiota by location: Beta diversity of the gut microbiota of children living in rural or urban locations in all intervention arms and at all ages was assessed using principal coordinates analysis (PCoA) of weighted Unifrac distances; Figure S2. Beta diversity of the gut microbiota by intervention arm: Beta diversity of the gut microbiota of children in all three intervention arms, living in rural or urban locations, and at all ages was assessed using principal coordinates analysis (PCoA) of weighted Unifrac distances; Figure S3. Relative abundance of major phyla: The relative abundance of the major taxa of the gut microbiota was assessed in QIIME Version 1.5. The relative abundance of the four major phyla, Actinobacteria, Proteobacteria, Firmicutes, and Bacteroidetes, of all children at each age group is shown; Figure S4. Relative abundance of the most abundant genera: The relative abundance of the gut microbiota was assessed in QIIME Version 1.5. The relative abundance of the 10 most abundant genera, *Bifidobacterium*, *Escherichia*/*Shigella*, *Lactobacillus*, *Streptococcus*, *Prevotella*, *Actinobacillus*, *Fecalibacterium*, *Klebsiella*, and *Megasphera Lachnospiraceae*, at each age group is shown. Table S1: Clinical data of children in urban and rural locations in each intervention arm at 3 monthly intervals; Table S2: Differences in alpha diversity indices between children in urban and rural locations at each 3-monthly time point; Table S3: Differentially abundant taxa in the microbiota of children residing in urban and rural locations identified by LEfSe analysis at 3-monthly intervals; Table S4: Differentially abundant taxa in the microbiota of children receiving different interventions identified by LEfSe analysis at 3-monthly intervals.
