# Supplementary material for: Longitudinal Analysis of the Intestinal Microbiota among a Cohort of Children in Rural and Urban Areas of Pakistan
Source: Nutrients. 2023 Feb 28;15(5):1213. doi: 10.3390/nu15051213 (PMC10005232; doi:10.3390/nu15051213)
Supplement: Supplementary file 1 [file nutrients-15-01213-s001.zip › nutrients-2114447-supplementary.pdf]

Table S1: Clinical data of children in urban and rural locations in each intervention arm at 3 monthly intervals

Parameter	Intervention Group			p-value	Residence		p-value
	A	B	C		Urban	Rural	
	n = 20	n = 20	n = 20		n = 30	n = 30	
**Gender**							
**Male**	7 (35.0)	6 (30.0)	10 (50.0)	0.502	10 (33.3)	13 (43.3)	0.596
**Female**	13 (65.0)	14 (70.0)	10 (50.0)		20 (66.7)	17 (56.7)	
**At Recruitment (3 months)**							
**Height (cm)**	61.0 (58.3,63.0)	59.0 (56.5,63.5)	59.0 (55.8,61.0)	0.190	61.2 (59.5,64.0)	58.3 (56.0,59.5)	<0.001
**Weight (kg)**	5.3 (4.3,5.6)	5.3 (4.5,5.9)	5.2 (4.5,5.6)	0.880	5.5 (5.1,6.0)	4.6 (4.2,5.3)	<0.001
**HAZ**	0.2 (-0.4,1.2)	-0.9 (-1.9,0.7)	-1.2 (-1.8,-0.1)	0.036	0.4 (-1.2,1.4)	-1.1 (-1.8,-0.2)	0.006
**WHZ**	-2.5 (-3.2,-1.4)	-1.3 (-2.3,-0.1)	-1.2 (-1.9,-0.3)	0.027	-1.9 (-2.6,-0.6)	-1.3 (-2.4,-0.9)	0.730
**WAZ**	-1.2 (-2.7,-0.4)	-1.4 (-2.2,-0.4)	-1.5 (-2.1,-1.1)	0.900	-1.1 (-1.7,-0.2)	-1.9 (-2.6,-1.3)	0.002
**Stunted (HAZ<-2SD)**	2 (10.0)	7 (35.0)	4 (21.1)	0.160	6 (20.7)	7 (23.3)	1.000
**Wasted (WHZ<-2SD)**	13 (65.0)	6 (30.0)	4 (21.1)	0.013	12 (41.4)	11 (36.7)	0.790
**Underweight (WAZ<-2SD)**	8 (40.0)	5 (25.0)	8 (40.0)	0.560	7 (23.3)	14 (46.7)	0.100
**Diarrhea**	3 (15.0)	5 (25.0)	8 (40.0)	0.240	4 (13.3)	12 (40.0)	0.039
**Micronutrient status**							
**Anemia (HB<**11 g/dL**)**	13 (68.4)	12 (66.7)	10 (50.0)	0.440	20 (71.4)	15 (51.7)	0.180
**Vitamin A deficiency (Retinol<**0·7 mmol/L**)**	3 (15.8)	4 (25.0)	7 (41.2)	0.260	7 (26.9)	7 (26.9)	1.000
**Iron deficiency (Ferritin<**12 ng/mL**)**	0 (0.0)	0 (0.0)	0 (0.0)	--	0 (0.0)	0 (0.0)	--
**Zinc deficiency (zinc<**60 μg/dL**)**	2 (10.5)	2 (12.5)	6 (35.3)	0.150	3 (11.5)	7 (26.9)	0.290
**At 6 months**							
**Height (cm)**	65.5 (63.5,67.3)	64.0 (61.2,68.0)	65.5 (62.0,68.0)	0.480	65.9 (64.0,68.0)	63.8 (61.1,67.0)	0.043
**Weight (kg)**	6.4 (5.7,7.7)	6.6 (6.0,7.2)	6.2 (5.6,6.8)	0.620	6.6 (5.9,7.5)	6.2 (5.4,6.9)	0.079
**HAZ**	-0.1 (-1.0,0.5)	-1.0 (-2.1,0.7)	-0.8 (-1.7,0.1)	0.430	0.0 (-1.0,0.5)	-1.2 (-2.1,0.3)	0.060
**WHZ**	-1.8 (-2.4,-0.3)	-1.1 (-1.7,-0.3)	-1.8 (-2.2,-1.0)	0.230	-1.2 (-2.0,-0.7)	-1.4 (-2.2,-0.5)	0.500
**WAZ**	-1.2 (-2.1,-0.0)	-1.0 (-2.0,-0.2)	-1.9 (-2.4,-1.2)	0.330	-1.1 (-2.0,-0.1)	-2.0 (-2.6,-0.8)	0.021
**Stunted (HAZ<-2SD)**	3 (15.8)	5 (26.3)	4 (21.1)	0.920	2 (6.9)	10 (35.7)	0.010
**Wasted (WHZ<-2SD)**	7 (36.8)	2 (10.5)	7 (36.8)	0.140	6 (20.7)	10 (35.7)	0.250
**Underweight (WAZ<-2SD)**	8 (40.0)	4 (20.0)	12 (63.2)	0.026	10 (33.3)	14 (48.3)	0.290

**Diarrhea**	8 (40.0)	5 (25.0)	7 (35.0)	0.700	10 (33.3)	10 (33.3)	1.000
**Micronutrient status**							
**Anemia (HB<**11 g/dL**)**	11 (64.7)	10 (71.4)	6 (40.0)	0.250	11 (57.9)	16 (59.3)	1.000
**Vitamin A deficiency (Retinol<**0·7 mmol/L**)**	3 (18.8)	4 (30.8)	3 (17.6)	0.740	3 (15.8)	7 (25.9)	0.490
**Iron deficiency (Ferritin<**12 ng/mL**)**	7 (53.8)	7 (58.3)	8 (50.0)	0.930	9 (56.3)	13 (52.0)	1.000
**Zinc deficiency (zinc<**60 µg/dL**)**	3 (18.8)	3 (23.1)	1 (5.9)	0.350	1 (5.3)	6 (22.2)	0.210
**At 9 months**							
**Height (cm)**	69.2 (67.5,70.5)	69.5 (67.0,72.0)	68.6 (67.5,71.0)	0.680	69.8 (68.5,71.9)	68.3 (65.0,71.0)	0.024
**Weight (kg)**	7.5 (6.2,8.0)	7.4 (6.3,7.9)	7.1 (6.3,8.0)	0.920	7.4 (6.7,7.9)	7.2 (6.1,7.9)	0.430
**HAZ**	-0.6 (-1.5,-0.1)	-0.3 (-1.7,0.6)	-0.9 (-1.7,-0.1)	0.470	-0.2 (-1.0,0.4)	-0.9 (-2.3,-0.4)	0.012
**WHZ**	-0.9 (-2.0,-0.6)	-1.3 (-2.6,-0.4)	-1.0 (-2.4,-0.2)	0.570	-1.1 (-1.9,-0.5)	-1.1 (-2.2,-0.3)	0.910
**WAZ**	-1.4 (-2.5,-0.4)	-0.9 (-2.2,-0.6)	-1.4 (-2.6,-0.8)	0.820	-1.3 (-2.0,-0.5)	-1.5 (-2.9,-0.8)	0.25
**Stunted (HAZ<-2SD)**	2 (12.5)	3 (20.0)	3 (20.0)	0.790	0 (0.0)	8 (30.8)	0.006
**Wasted (WHZ<-2SD)**	4 (25.0)	4 (26.7)	4 (26.7)	1.000	4 (20.0)	8 (30.8)	0.510
**Underweight (WAZ<-2SD)**	6 (30.0)	6 (31.6)	10 (52.6)	0.290	9 (32.1)	13 (43.3)	0.430
**Diarrhea**	5 (25.0)	8 (40.0)	11 (55.0)	0.170	8 (26.7)	16 (53.3)	0.064
**At 12 months**							
**Height (cm)**	70.0 (67.5,74.0)	71.3 (69.0,73.0)	71.5 (68.0,73.0)	0.990	71.5 (69.0,74.0)	70.0 (66.0,73.0)	0.180
**Weight (kg)**	7.9 (6.6,8.6)	7.8 (7.2,8.5)	7.7 (7.0,8.0)	0.760	7.9 (7.1,8.5)	7.6 (6.5,8.4)	0.430
**HAZ**	-1.7 (-3.0,0.0)	-1.5 (-2.8,-0.7)	-1.6 (-2.5,-0.7)	0.940	-1.4 (-2.0,-0.4)	-2.0 (-3.1,-0.7)	0.210
**WHZ**	-1.0 (-2.3,-0.6)	-0.6 (-1.7,-0.2)	-1.2 (-2.2,-0.2)	0.630	-0.7 (-1.5,-0.4)	-1.7 (-2.3,-0.2)	0.210
**WAZ**	-1.6 (-2.6,-0.4)	-1.2 (-2.2,-0.7)	-1.8 (-2.8,-0.9)	0.430	-1.2 (-2.3,-0.6)	-1.8 (-2.6,-0.9)	0.270
**Stunted (HAZ<-2SD)**	5 (35.7)	4 (30.8)	4 (40.0)	0.910	5 (27.8)	8 (42.1)	0.500
**Wasted (WHZ<-2SD)**	6 (42.9)	3 (23.1)	3 (30.0)	0.620	3 (16.7)	9 (47.4)	0.079
**Underweight (WAZ<-2SD)**	7 (35.0)	5 (25.0)	9 (47.4)	0.360	10 (34.5)	11 (36.7)	1.000
**Diarrhea**	8 (40.0)	4 (20.0)	8 (40.0)	0.340	5 (16.7)	15 (50.0)	0.013
**At 18 months**							
**Height (cm)**	76.5 (73.0,78.0)	77.0 (75.0,79.0)	79.0 (75.0,79.0)	0.480	78.5 (76.5,79.0)	75.9 (73.0,78.5)	0.150
**Weight (kg)**	9.0 (7.7,9.7)	8.8 (8.3,9.8)	8.4 (7.6,9.9)	0.830	9.8 (8.6,10.1)	8.4 (7.5,9.3)	0.013
**HAZ**	-1.6 (-3.4,-0.9)	-1.3 (-2.6,-0.6)	-1.2 (-2.3,-1.0)	0.750	-1.2 (-1.6,-0.7)	-1.9 (-3.2,-1.2)	0.120
**WHZ**	-0.5 (-1.1,0.7)	-0.5 (-1.3,0.1)	-0.7 (-1.9,-0.2)	0.630	-0.1 (-1.1,0.3)	-0.9 (-1.7,-0.4)	0.026
**WAZ**	-1.1 (-2.4,-0.4)	-1.2 (-2.2,-0.3)	-1.7 (-2.5,-0.4)	0.640	-0.6 (-1.7,-0.3)	-2.0 (-2.7,-1.1)	0.004
**Stunted (HAZ<-2SD)**	2 (22.2)	3 (27.3)	4 (28.6)	1.000	4 (23.5)	5 (29.4)	1.000

**Wasted (WHZ<-2SD)**	1 (11.1)	1 (9.1)	3 (21.4)	0.840	2 (11.8)	3 (17.6)	1.000
**Underweight (WAZ<-2SD)**	6 (35.3)	6 (30.0)	12 (63.2)	0.093	9 (34.6)	15 (50.0)	0.290
**Diarrhea**	8 (40.0)	4 (20.0)	7 (35.0)	0.470	9 (30.0)	10 (33.3)	1.000
**Micronutrient status**							
**Anemia (HB<**11 g/dL**)**	18 (94.7)	11 (57.9)	13 (68.4)	0.025	19 (67.9)	23 (79.3)	0.38
**Vitamin A deficiency (Retinol<**0·7 mmol/L**)**	6 (35.3)	8 (47.1)	9 (50.0)	0.690	16 (64.0)	7 (25.9)	0.011
**Iron deficiency (Ferritin<**12 ng/mL**)**	6 (75.0)	1 (6.3)	5 (33.3)	0.002	3 (17.6)	9 (40.9)	0.17
**Zinc deficiency (zinc<**60 μg/dL**)**	7 (38.9)	4 (21.1)	5 (27.8)	0.500	0 (0.0)	16 (53.3)	<0.001
**At 24 months**							
**Height (cm)**	79.0 (77.0,82.0)	79.8 (77.5,82.5)	80.0 (77.5,82.7)	0.850	81.0 (78.0,82.7)	79.0 (76.8,82.0)	0.200
**Weight (kg)**	9.4 (8.0,10.6)	9.1 (8.4,10.3)	9.4 (8.3,9.8)	0.990	9.6 (9.0,10.8)	8.8 (7.8,9.8)	0.043
**HAZ**	-2.3 (-3.1,-1.7)	-2.1 (-2.9,-1.3)	-2.1 (-2.8,-1.2)	0.950	-1.8 (-2.8,-1.1)	-2.4 (-3.2,-1.7)	0.140
**WHZ**	-1.2 (-1.9,-0.9)	-0.7 (-1.8,-0.4)	-1.1 (-1.8,-0.7)	0.810	-0.9 (-1.1,-0.5)	-1.5 (-1.9,-0.6)	0.120
**WAZ**	-2.0 (-3.2,-0.7)	-1.9 (-3.1,-1.3)	-1.9 (-3.1,-1.4)	0.910	-1.6 (-2.1,-0.8)	-2.4 (-3.3,-1.6)	0.013
**Stunted (HAZ<-2SD)**	8 (57.1)	9 (56.3)	9 (52.9)	1.000	8 (42.1)	18 (64.3)	0.150
**Wasted (WHZ<-2SD)**	3 (21.4)	2 (12.5)	3 (17.6)	0.890	2 (10.5)	6 (21.4)	0.440
**Underweight (WAZ<-2SD)**	11 (57.9)	9 (47.4)	10 (52.6)	0.940	11 (40.7)	19 (63.3)	0.110
**Diarrhea[3]**	1 (5.0)	1 (5.0)	4 (20.0)	0.340	2 (6.7)	4 (13.3)	0.670

[1] *For categorical variables summary statistics reflect the count and percent in parenthesis; the difference was assessed with Fishers exact test*

[2] *For continuous variables summary statistics are presented as median and IQR; the difference was assessed with the Mann-Whitney test*

[3] *Diarrhea was defined as three or more loose stools per day. A diarrheal episode was defined as a minimum of 2 days with diarrhea followed by at least 2 diarrhea-free days.*

Table S2: Differences in alpha diversity indices between children in urban and rural locations at each 3-monthly time point

Index	Location	Month					
Chao		**3**	**6**	**9**	**12**	**18**	**24**
	Rural[1]	349.2 (293.0, 469.0)	443.2 (376.5, 514.8)	478.8 (411.7, 579.8)	462.2 (388.6, 599.3)	736.0 (589.6, 974.7)	767.8 (589.5, 973.9)
	Urban[1]	444.9 (345.9, 520.9)	458.6 (369.1, 573.4)	429.8 (361.6, 533.2)	521.3 (438.4, 677.8)	634.9 (408.6, 894.0)	811.0 (660.3, 1113.7)
	P-value[2]	0.1421	0.7117	0.2665	0.2488	0.1462	0.4581
Shannon							
	Rural	2.75 (2.20, 3.31)	3.14 (2.82, 3.57)	3.58 (3.21, 3.98)	3.62 (3.14, 3.92)	4.51 (3.63, 4.89)	4.85 (4.02, 5.30)
	Urban	3.25 (2.39, 3.71)	3.39 (3.03, 4.04)	3.26 (2.58, 3.82)	3.72 (3.20, 4.24)	4.13 (2.96, 4.93)	5.00 (4.25, 5.45)
	P-value	0.1153	0.1296	0.2009	0.1433	0.3671	0.3554
Equitability							
	Rural	0.374 (0.324, 0.440)	0.424 (0.383, 0.472)	0.472 (0.423, 0.511)	0.478 (0.423, 0.503)	0.552 (0.469, 0.583)	0.589 (0.510, 0.618)
	Urban	0.433 (0.354, 0.488)	0.454 (0.418, 0.509)	0.432 (0.363, 0.510)	0.484 (0.434, 0.531)	0.523 (0.401, 0.588)	0.596 (0.527, 0.639)
	P-value	0.0713	0.1188	0.1785	0.1205	0.3632	0.3671
PD							
	Rural	20.00 (15.29, 24.36)	22.74 (20.27, 25.15)	24.34 (21.96, 29.98)	26.91 (21.91, 30.75)	37.69 (30.86, 42.82)	39.52 (33.97, 47.34)
	Urban	22.50 (18.02, 30.26)	24.75 (19.86, 30.49)	24.95 (21.97, 28.85)	28.38 (24.17, 34.18)	32.58 (25.14, 41.80)	40.49 (35.74, 45.36)
	P-value	0.0850	0.3898	0.8891	0.1984	0.1882	0.9176
Observed							
	Rural	149.5 (119.2, 187.8)	165.5 (152.0, 192.5)	192.0 (174.2, 232.5)	190.5 (155.5, 230.5)	271.0 (212.0, 331.8)	292.5 (242.5, 378.5)
	Urban	172.5 (113.8, 200.8)	187.5 (145.8, 229.8)	181.5 (148.2, 226.5)	201.5 (178.5, 263.2)	248.0 (163.2, 320.2)	317.0 (271.5, 388.0)
	P-value	0.3750	0.2169	0.2642	0.09477	0.2549	0.4917

[1]median (interquartile range), [2]Mann-Whitney

Table S3: Differentially abundant taxa in the microbiota of children residing in urban and rural locations identified by LEfSe analysis at 3-monthly intervals

Urban	Rural
**3 months**	
Clostridiaceae	None
Clostridiaceae_1	
Clostridium	
Lachnospiraceae	
Lachnospiraceae (subfamily)	
Lachnospira	
Lachnospiraceae_Incertae_Sedis	
Lactococcus	
Corynebacteriaceae	
Corynebacterium	
Alphaproteobacteria	
**6 months**	
Firmicutes	Actinobacteria
Clostridiaceae_1	Actinobacteria (class)
Clostridiaceae	Actinobacteridae
Clostridium	Bifidobacteriales
Enhydrobacter	Bifidobacteriaceae
Incertae_Sedis_6	Bifiobacterium
Peptoniphilus	
**9 months**	
None	Actinobacillus
	Pasteurellaceae
	Pasteurellales
**12 months**	
Veillonellaceae	
**18 months**	
Enterococcaceae	Catenibacterium
Enterococcus	Erysipelotrichi
Ruminococcaceae	Erysipelotrichales
Ruminococcaceae_Incertae_Sedis	Erysipelotrichaceae
**24 months**	
Sporobacter	Clostridiaceae
	Clostridiaceae_1
	Clostridium
	Cyanobacteria
	Streptophyta
	Chloroplast
	Cyanobacteria (class)
	Leuconostoc
	Sarcina

Table S4: Differentially abundant taxa in the microbiota of children receiving different interventions identified by LEfSe analysis at 3-monthly intervals

Intervention Group	A	B	C
**3 months**			
	None	None	None
**6 months**			
	None	None	Actinomyces<br>Actinomycetaceae<br>Actinomycineae<br>*Atopobium*
**9 months**			
	None	None	*Dialister*
**12 months**			
	None	None	*Weisella*
**18 months**			
	None	None	None
**24 months**			
	None	None	None

Supplementary Figures

Figure S1

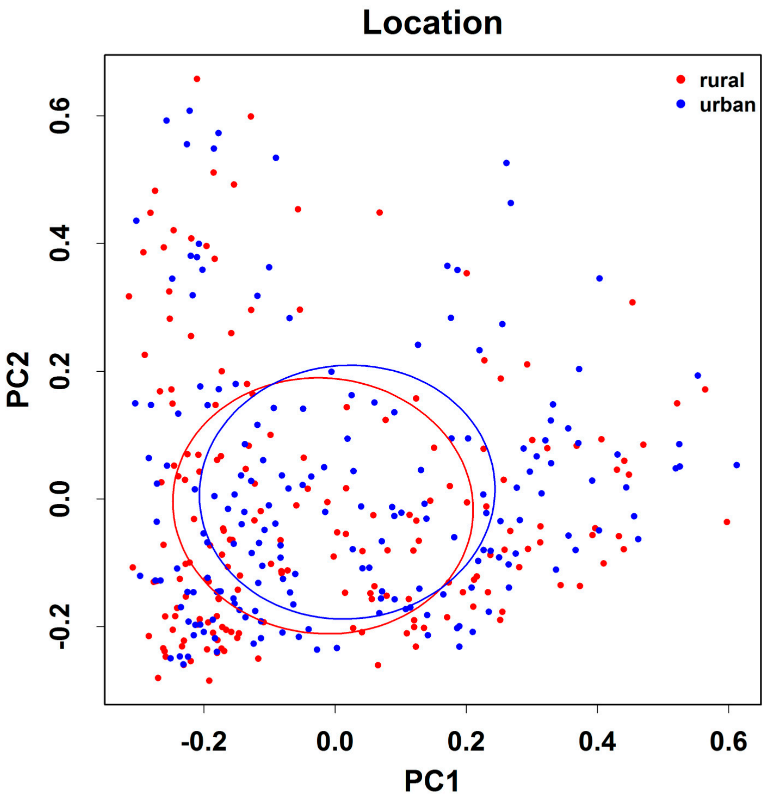

Figure S2

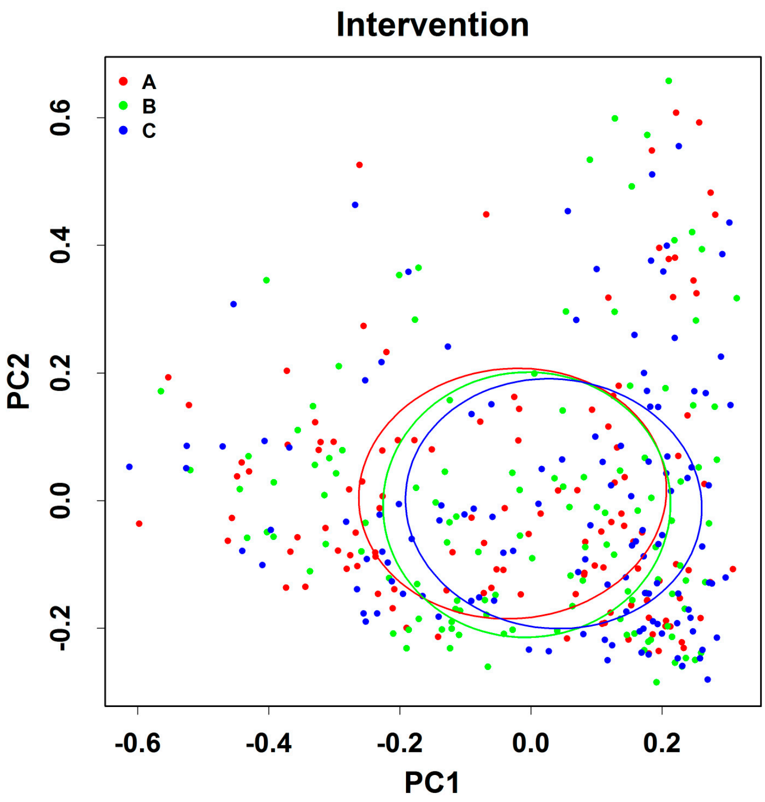

Figure S3

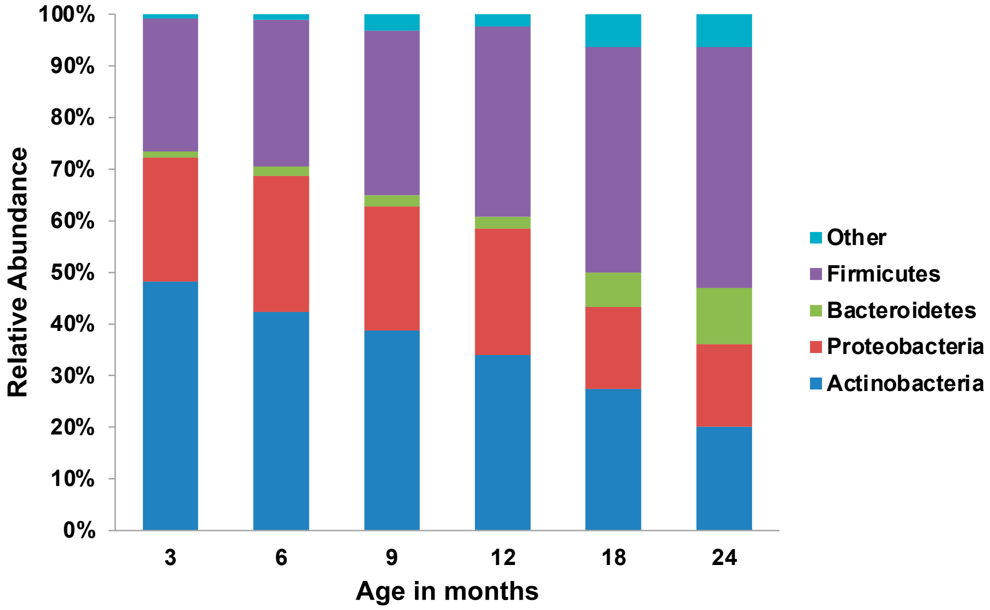
100%
90%
80%
70%
60%
50%
40%
30%
20%
10%
0%
Relative Abundance
3
6
9
12
18
24
Age in months
Other
Firmicutes
Bacteroidetes
Proteobacteria
Actinobacteria

Figure S4

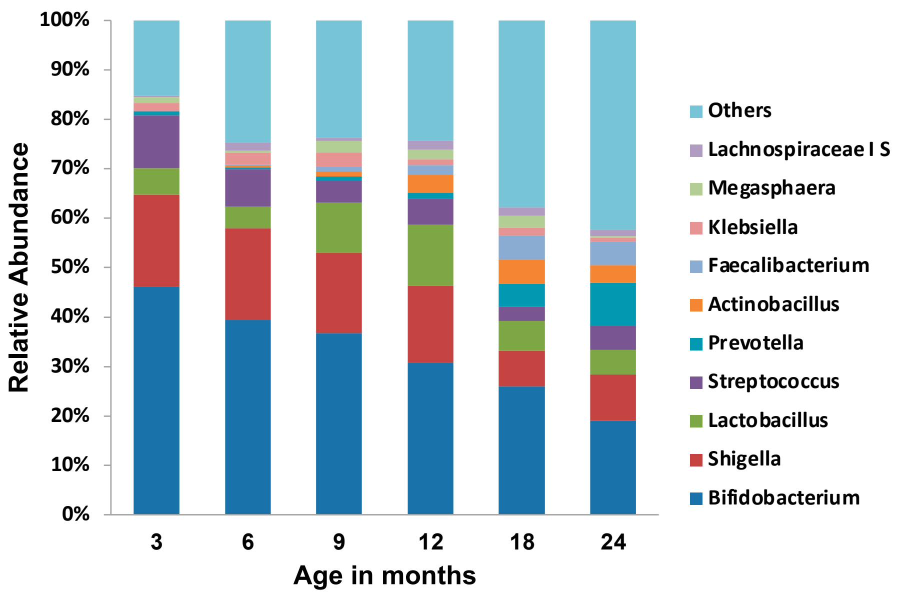
100%
90%
80%
70%
60%
50%
40%
30%
20%
10%
0%
Relative Abundance
3
6
9
12
18
24
Age in months
Others
Lachnospiraceae I S
Megasphaera
Klebsiella
Faecalibacterium
Actinobacillus
Prevotella
Streptococcus
Lactobacillus
Shigella
Bifidobacterium
